# A health knowledge brokering intervention in a district of Burkina Faso: A qualitative retrospective implementation analysis

**DOI:** 10.1371/journal.pone.0220105

**Published:** 2019-07-26

**Authors:** Esther Mc Sween-Cadieux, Christian Dagenais, Donmozoun Télesphore Somé, Valéry Ridde

**Affiliations:** 1 Department of Psychology, University of Montreal, Montreal, Quebec, Canada; 2 Agence de Formation de Recherche et d’Expertise en Santé pour l’Afrique (AFRICSanté), Bobo-Dioulasso, Burkina Faso; 3 French Institute for Research on Sustainable Development (IRD), CEPED (IRD-Université Paris Descartes), Universités Paris Sorbonne Cités, ERL INSERM SAGESUD, Paris, France; 4 University of Montreal Public Health Research Institute (IRSPUM), Montreal, Quebec, Canada; Ghana Health Services, GHANA

## Abstract

**Background:**

A knowledge brokering (KB) intervention was implemented in Burkina Faso. By creating partnerships with health system actors in one district, the broker was expected to assess their knowledge needs, survey the literature to provide the most recent research evidence, produce various knowledge translation tools, and support them in using research to improve their actions. The purpose of this study was to analyze the key factors that influenced the KB project and to make recommendations for future initiatives.

**Methods:**

The qualitative design involved a single case study in which the KB intervention implementation was evaluated retrospectively. Data came from interviews with the intervention team (n = 4) and with various actors involved in the intervention (n = 16). Data from formative evaluations conducted during the KB implementation and observation data from a two-month field mission were also used. Two conceptual frameworks were combined to guide the analysis: the *Consolidated Framework for Implementation Research* (Damschroder et al., 2009) and the *Ecological Framework* (Durlak & DuPre, 2008).

**Results:**

Various KB activities were conducted during the first two years of implementation at the local level. The project came to an early end following vain efforts to relocate the intervention at the central level in order to further influence the policy process. Certain shortcomings in the implementation team negatively influenced the implementation: inadequate leadership, no shared vision regarding the reorientation of the intervention, challenges related to the KB role, and lack of frank communications internally. Other impediments to the intervention’s deployment included local actors' lack of decision-making authority, the unavailability of resources and of organizational incentives for involvement in the KB intervention, and contextual challenges in accessing the central level. However, the KB strategy presented several strengths: collaborative development, support provided to local partners by the broker, and training opportunities and support provided to the broker.

**Conclusions:**

More attention must be paid to intervention planning, partners’ engagement, human, financial and technical resources availability, continuous development of skills and of communications within the KB team, and periodic assessment of potential obstacles related to the complexity of the system within which the intervention has been implemented. Using implementation science frameworks when developing KB strategies in the West African context should be promoted.

## Introduction

In global health, the need to make research-based evidence (RBE) available to inform health practices and public policy requires no further demonstration [1]. Health system actors in low-income countries are encouraged to rely as much as possible on such evidence when developing health policies to improve population health indicators [2,3]. As such, knowledge translation (KT) strategies are increasingly aimed at improving the accessibility and practical relevance of research results and providing RBE at the right time in the decision-making process [4]. However, there are not yet many KT initiatives being implemented and assessed on the African continent [5].

### Knowledge brokering

Knowledge brokers are recognized as having a role to play in creating links between researchers, practitioners, and decision-makers. Knowledge brokering (KB) has been defined as a KT strategy that “links researchers and decision-makers, facilitating their interaction so that they are better able to understand each other’s goals and professional culture, influence each other’s work, forge new partnerships and use research-based evidence” [6]. The objective of these interactions is also to mobilize RBE in combination with actors’ experiential knowledge and to foster its use [7,8].

The most commonly used KB activities are: acquiring and adapting RBE, using various tools to disseminate RBE, and developing networks and partnerships [9,10]. Knowledge brokering is a means of establishing collaborative links between researchers and potential users of RBE, identifying issues for which solutions are needed, and finding and retrieving the relevant RBE to meet those needs. The broker can then interpret the evidence in light of the various actors’ knowledge so that it can be translated into action [9,11]. Knowledge brokers also prepare evidence dissemination tools that are attractive and tailored to the target audiences [12]. The broker’s role is not limited to acquiring, adapting, and disseminating information, but also involves providing ongoing support for change [13,14]. Another KB function often mentioned [15] but rarely applied [9] is strengthening actors’ capacities in terms of acquiring, assessing, interpreting, and applying evidence and developing KT strategies [11,16]. Knowledge brokering strategies also need to be tailored [17] to the objectives and needs of the actors involved [9] and be appropriate to the context [11,15].

A recent systematic review concluded there is not yet sufficient evidence on the efficacy of interventions that rely on researcher–user interactions to influence RBE use [18]. However, positive outcomes of KB have been observed in a few studies [19–22], one of which found that knowledge brokers had a greater impact in organizations with a less well-developed culture of evidence use [11]. While studies to assess the efficacy of KT strategies are essential, analyses of implementation processes are still needed to understand why and how those strategies succeed or fail [23,24]. Despite the recent proliferation of KT conceptual frameworks [25–27], the transition from theory to practice remains a KT challenge [28].

### A knowledge brokering intervention in Burkina Faso

In 2012, a KB intervention was developed as part of a major interventional research program in a health district of Burkina Faso. The strategy was first implemented in a rural district, not far from the capital (100 km), which represents one of the major urban centers of the country. The research project was a partnership between Canada and Burkina Faso, funded by the Canadian Institutes of Health Research. The KB objectives were to 1) provide health actors in the intervention district with RBE to meet their needs, and 2) foster the transfer and application of knowledge generated by a team of researchers involved in the research program on malaria control, maternal health, health financing, and mutual health organizations (MHO). The role of the broker was therefore to act as a liaison between knowledge producers and potential users of research data (e.g. mutual health organizations, non-governmental organizations, health practitioners, decision makers). This KB intervention was implemented to counteract the difficulty in accessing, understanding and using research-based evidence, often identified by the health actors during previous project [29]. Hence a local knowledge broker was recruited, supported by a senior broker onsite and by several KT specialists and researchers in Canada, including two co-authors of this article. The broker underwent several training sessions to strengthen his KT capacities: two 5-day sessions in 2012 and a 2-week observation internship in Canada with KB specialists. The content of the training sessions is presented elsewhere [30].

The intervention was developed based on the latest knowledge available at that time [31]. The activities that were planned were similar to those currently defined in the literature [9,13,32]. Furthermore, the intervention was validated in collaboration with partners (RBE users) in the field during two days of consultation to ensure it was adapted to their knowledge needs and to the local context. The objective of this consultation was to clarify how KB could be useful and how it could be integrated into the activities of the various health actors and organizations in the intervention district. For example, the actors raised several questions to which knowledge was already available in the scientific literature (e.g., What factors explain the low public adherence to mutual health organizations? What consciousness-raising methods have positive impacts on public awareness?). It was expected that the broker undertakes several KT activities such as knowledge management and literature review, development of KT tools adapted to RBE users and organization of dissemination workshops as well as ensuring a follow-up with users. The KB activities were conducted in French, but the broker also had a very good knowledge of English to assess and translate research-based evidence.

**Fig 1:**
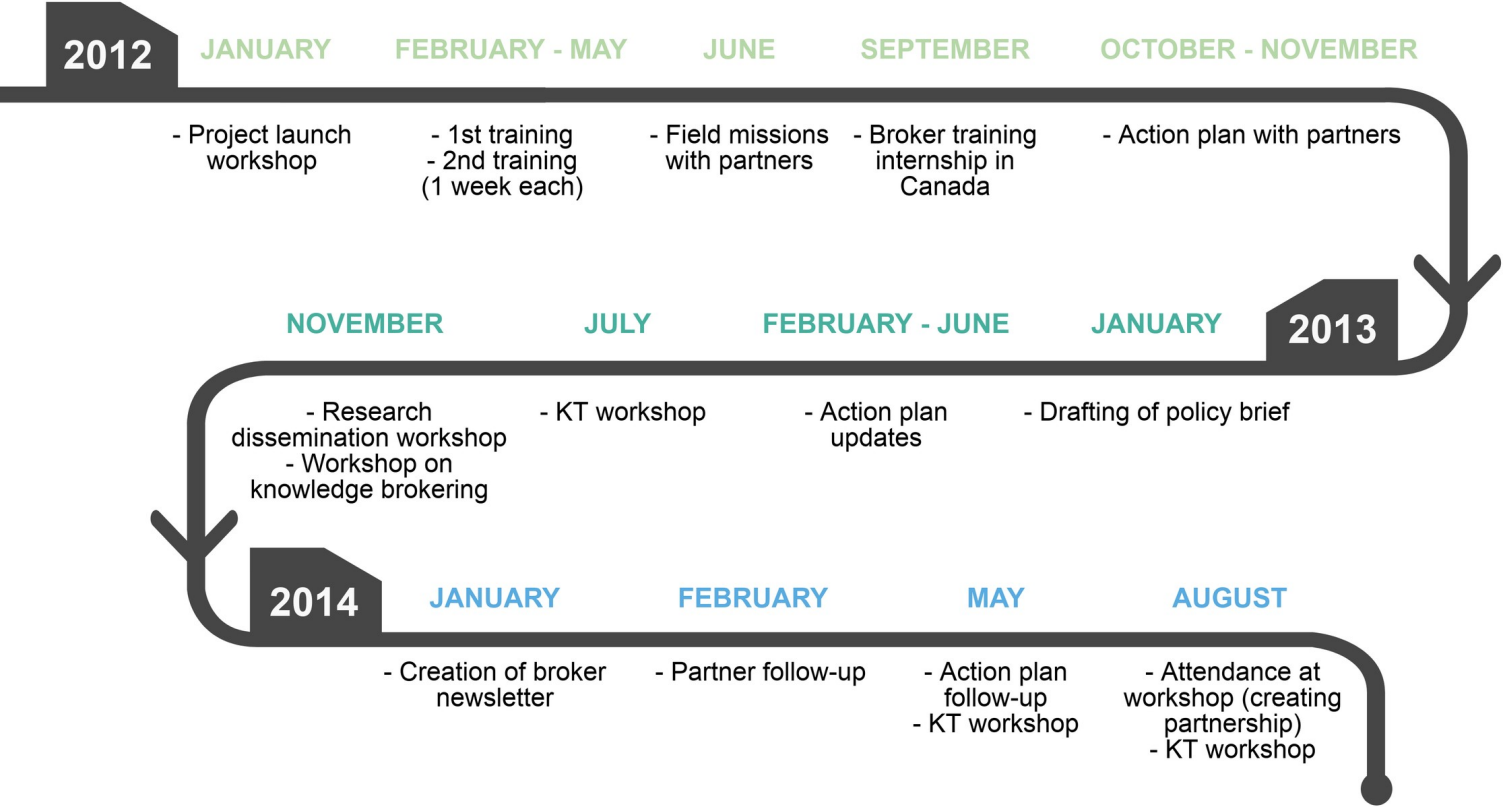
Chronology of the intervention. The main knowledge brokering activities carried out during the three years of implementation (2012 to 2014) of the intervention are summarily described.

An evaluation conducted one year after the start of implementation showed that the broker’s activities were seen as being very helpful for improving health practices, especially by RBE users at the local level (e.g., MHOs and NGOs in the district) [30]. Fig 1 illustrates the main activities conducted during the three years of implementation, which were numerous: meeting with partners, surveying the literature, drafting policy briefs, preparing and disseminating a monthly newsletter for partners, and organizing workshops to share knowledge acquired from the surveys and evidence being produced by the research program [see S1 Table]. During the first year, the brokering activities reached more than 50 actors from various sectors such as research institutes, the Ministry of Health, local and regional authorities, non-governmental organizations (NGOs), and MHOs. For example, the knowledge broker supported mutual health organizations for almost two years. Together they developed an action plan, based on context-adapted research results, to increase membership in mutuals. Upon applying that plan and after several follow-up meetings with the broker, the different MHOs saw increases in their membership after one year [33]. However, the activities came to an early end during the implementation’s third year, following efforts to relocate the intervention at the central level in order to further influence the political sphere (e.g. Ministry of Health, a national research center). For more information on the development and the KB intervention, see Dagenais et al., (2015) [30].

The objectives of this study are to: 1) analyze the factors that influenced the process of implementing the KB intervention; and 2) propose recommendations for the development of a future KB intervention.

## Methods

**Fig 2:**
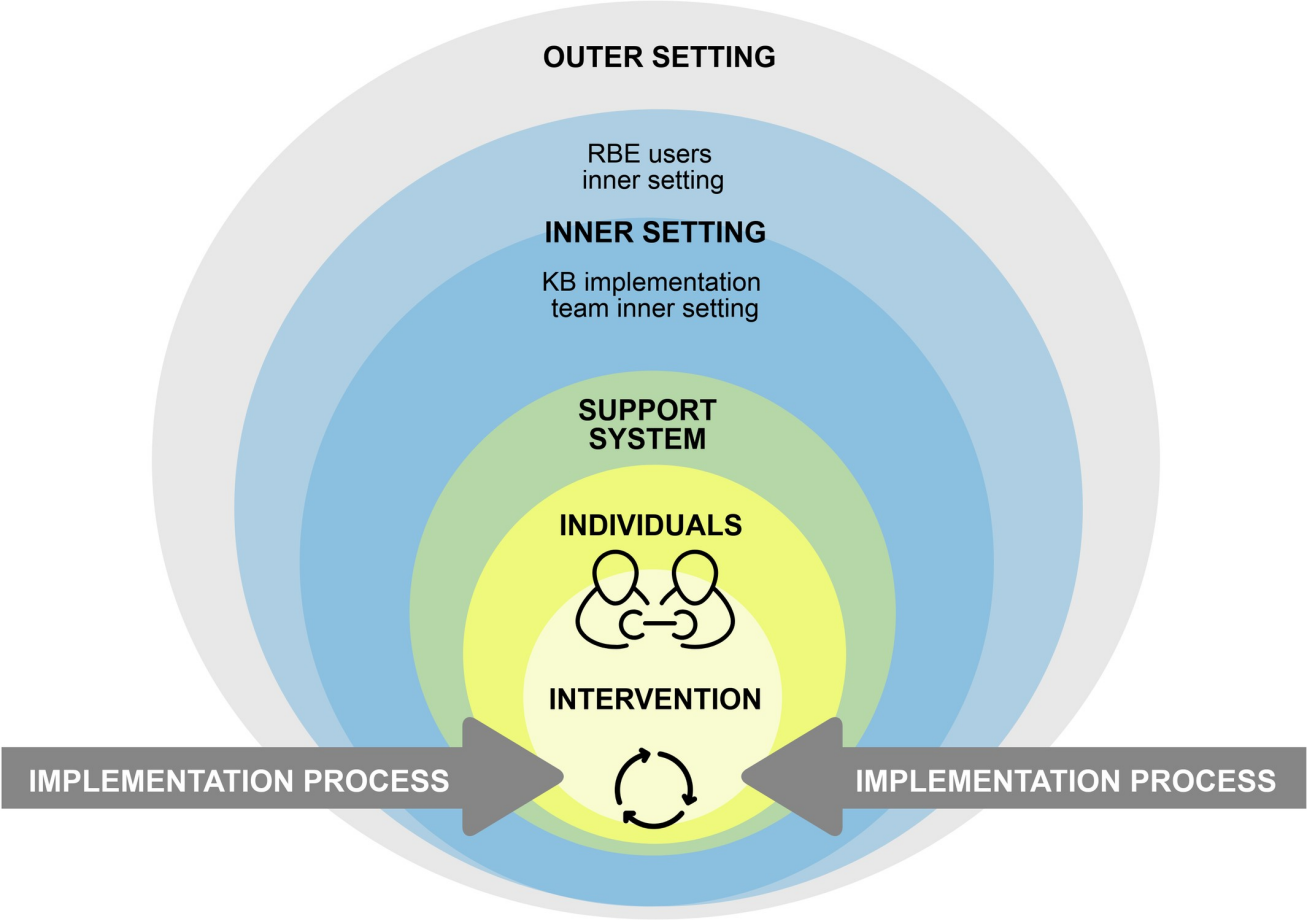
Conceptual framework. The six dimensions illustrate each level of analysis of the knowledge brokering intervention.

This implementation analysis used a qualitative single case study because it allows to explain the complexities of real-life situations [34]. It can be defined as an intrinsic case study, according to Stake (1995) [35], because the intent of the case analysis is not to understand a generic phenomenon or to construct a theory, but to gain an in-depth understanding of the case. The case is the knowledge brokering intervention implemented in Burkina Faso between 2012 and 2014. It includes all the actors involved in the development and implementation: the knowledge brokers, the designers of the intervention, and the support team, as well as the strategy partners who collaborated with the broker. The organizational, social and political contexts in which the intervention took place are also part of this case study analysis. The case boundaries are shown in Fig 2. This case study was both descriptive and explanatory in order to examine over time the intervention’s implementation and to understand the factors that have influenced it [34]. These factors were analyzed retrospectively following the termination of the intervention.

### Conceptual framework

To gain a comprehensive understanding of the implementation [27], two conceptual frameworks were combined: the Ecological Framework (EF) of Durlak and DuPre (2008) [36] and the Consolidated Framework for Implementation Research (CFIR) of Damschroder et al. (2009) [37]. These frameworks are used to identify retrospectively the factors that influence an intervention’s implementation [27]. The factors proposed by these authors are associated with good-quality implementation based on implementation theories and empirical studies. Both frameworks are based on a systems approach and cover a wide spectrum of factors at multiple levels (e.g. individual, intervention, context, process) that are essential for analyzing the knowledge brokering strategy. The EF is used more as a complement to the CFIR, as it focuses on a level of analysis that is less present in the CFIR, which has to do with factors related to the intervention’s support system.

#### Dimensions of the knowledge brokering intervention

The two frameworks combined covered the six dimensions of analysis illustrated in Fig 2. The intervention being analyzed is a knowledge brokering strategy in public health (1-KB INTERVENTION) that involved a junior knowledge broker and a senior broker who was supposed to facilitate brokering activities on the ground. They were based in a private research and evaluation consulting firm, and the activities were conducted with Canadian researchers who had co-designed the intervention (2-INDIVIDUALS). Another dimension consisted of factors related to the organizational context within which all these actors (brokers and support team) collaborated to carry out the brokering activities (3-INNER SETTING). Since the brokers were working in close collaboration with several health sector stakeholders (NGOs, health professionals, MHOs, decision-makers), those actors’ organizational contexts were also analyzed. These stakeholders represent the main targets of the intervention (also known as RBE users), and are defined in the article as the intervention partners. The intervention’s implementation was also supported and guided by KT specialists in Canada. This support system provided those doing the implementation with the skills required for its proper functioning, through training and the availability of technical support (4-SUPPORT SYSTEM). The factors related to Burkina Faso’s social and political context (5-OUTER SETTING) were also included in the framework. The final dimension of analysis focused on the activities carried out to facilitate the implementation process (6-IMPLEMENTATION PROCESS).

#### Underlying constructs of the different dimensions

Table 1 presents the constructs from the CFIR, along with factors added by incorporating the EF as a complementary conceptual framework (see S2 Table for more details on domains and constructs). As recommended, we have adapted them slightly to address our specific case. Some CFIR constructs were not retained as they did not apply to our case study (e.g. trialability, peer pressure).

**Table 1:** Domains and constructs from conceptual frameworks.

DOMAINS	CONSTRUCTS
1- INTERVENTION	Adaptability Intervention source Relative advantage Evidence strength and quality Design quality and packaging Complexity Cost
2- INDIVIDUALS	Knowledge and beliefs about the intervention Self-efficacy Individual identification with organization Skill proficiency
3- INNER SETTING	Structural characteristics Networks and communications Organizational culture Organizational norms regarding change Formulation of tasks Shared decision-making Implementation climate Tension for change Compatibility Relative priority Organizational incentives and rewards Goals and feedback Learning climate Readiness for implementation Leadership engagement Available resources Access to knowledge and information
4- SUPPORT SYSTEM	Training Technical assistance
5- OUTER SETTING	Needs and resources Cosmopolitanism External policy and incentives
6- IMPLEMENTATION PROCESS	Planning Executing Engaging Reflecting and evaluating

### Data sources

The data analyzed were drawn primarily from formative evaluations conducted during the first years of the intervention’s implementation [30,33]. Hence, interviews were conducted in 2013 with various health actors in the intervention district, which participated in KB activities (n = 16). The respondents came from various Ministry of Health agencies and local and regional authorities (health district, high commission, regional health department) (n = 5), local and international NGOs (mainly project coordinators) (n = 3), and local MHOs’ representatives (n = 8). Also included in the analysis were interviews with the two brokers conducted during the intervention (n = 2).

Observation data collected by the first author during a two-month field mission in July–August 2014 to accompany the knowledge broker during the activities were also used. A logbook was filled following various activities (meeting with the partners and debriefing, presentation of the intervention to decision-makers, knowledge brokering activities’ organization, meetings between the broker and the support team, etc.). These data included informal exchanges with local stakeholders, strategy partners and the KB team as well as contextual understanding elements. Moreover, data from critical analysis by persons involved in designing, supporting and implementing the intervention, who co-authored this article (CD, TS & VR), were also taken into account (e.g., during debriefing meetings). Thus, after the intervention was terminated (2016), interviews were conducted with members of the design and support team (n = 2).

As stated earlier, two co-authors (CD & VR) were part of the design and support team. They have previously published on or participated in projects that have examined issues with ineffectiveness and implementation challenges [29,38–40]. Thus, a reflexive approach was used in this study which can be defined as *« intended and conscious intellectual activity in which individuals (or groups) explore or examine their experiences to develop new understandings that ultimately shape their actions* » [41]. Consequently, it was recognized that this case study was not intended to put responsibility on individuals but to understand implementation mechanisms, thus allowing a critical reflection of the process. Finally, the first author, who was not directly involved in the implementation’s decisions, had the objective to arrive at a nuanced portrait of the intervention implementation based on all stakeholders' perspectives.

The protocol used for the interviews with the partners during implementation covered several elements such as the involvement of each actor in the intervention, their appreciation of the activities as well as the improvements proposed according to their experience. The post-intervention interviews were based on a grid developed from the conceptual frameworks and which covered the six domains described in Table 1 (e.g. intervention, individuals, support system, inner setting, outer setting and implementation process). The interviews lasted about 45 minutes on average. The interview guides can be found in S1 File.

### Data analysis

All interviews were transcribed, and NVivo11 software was used to facilitate the analysis. Qualitative data were analyzed thematically [42]. The conceptual frameworks were used to analyze, interpret, and report the results of the KB intervention implementation. All data were thus coded according to the six dimensions representing the themes (Fig 2), after which each segment was assigned one or more sub-themes representing the explanatory construct of each dimension (Table 1). The coding process was thus hierarchical, arranged from the broadest level of analysis to the most detailed. Given the large number of constructs to be considered, this technique facilitated coding and alleviated the cognitive burden associated with using the conceptual frameworks. Only the constructs with the greatest influence on the implementation process are reported in the results. On some occasions, reflective exchanges with external actors familiar with the context and the intervention were used to ensure a nuanced analysis of the data.

Ethics certificates were obtained from the research ethics committees of the University of Montreal Hospital Research Centre (12.273) and the National Health Ethics Committee of Burkina Faso (2012-11-85). Informed consent to participate was obtained from participants before interviews; the objective of the study was explained to them (i.e. to improve the KT intervention based on their experience) and the subsequent data utilisation. Confidentiality and anonymity have been assured to participants. Participants gave their verbal consent to audio record the interview and to participate in the study. Both consents were audio recorded. This knowledge translation study is part of a large research program that has been approved by both ethics committee in Burkina Faso and Canada.

## Results

The results are presented according to the six dimensions used to analyze the knowledge brokering intervention's implementation: intervention characteristics, the individuals involved, the inner setting, the support system, the outer setting and the implementation process (Fig 2).

### Intervention

Developing the strategy in collaboration with partners is a factor that facilitated implementation. This participatory process was seen as a strength of the intervention:

*“There was strong acceptance and receptivity*, *especially among field workers*. *The way the initiative was introduced was*, *without question*, *a key factor in strengthening the partnership*.*”* (Knowledge broker)

In two workshops with local actors, their perceptions and needs were assessed to ensure the strategy was tailored to the context (**adaptability**). The partners were enthusiastic and interested in the KB intervention as expressed by this respondent:

*“After the workshop on the KB intervention*, *I realized that we are potential beneficiaries, because it addresses different needs. (…) Each of us has seen that one day we may have to require the brokers’ services as part of our projects, to evaluate what we are doing or to guide us in what we want to do.” (NGO representative)*

However, in the post-intervention analysis, there were persistent questions about the extent to which the strategy was actually integrated into the partners’ organizational needs. As noted by this support team member, the fact that the resources provided by this project led by international researchers were available only for a limited time may have influenced the partners’ actual involvement:

*“[It remains to be seen] whether [the innovation] can satisfy a need and how it can be embedded into an organizational logic*, *to avoid reinforcing the perception that [international researchers] come with their ideas*, *do the project*, *and then leave*, *and nothing is sustained*.*”* (Support team member)

During the first year of implementation, stakeholders repeatedly spoke about the relevance of RBE in guiding their decisions. Several recognized the importance of the broker’s support in fuelling their reflection on their practices (**relative advantage**). However, this did not lead to any active and sustained partner involvement. As pointed out by a support team member, there was not enough communication to demystify the role that the broker could have played concretely with each partner. Moreover, because the intervention was an innovation, no evidence was yet available on the effectiveness of KB in improving health practices and policies (**evidence strength and quality**). An NGO representative, in fact, noted the importance of demonstrating to partners the positive impacts of brokering, to encourage their buy-in:

*“They [brokers] need to publish8 and disseminate in order to give visibility to what they’re doing*, *the impact they’re having in the field*, *and how that’s helping to improve the services of such-and-such an organization*. *That could be more persuasive for the partners*.*”* (NGO representative and intervention partner)

At the two workshops held while planning the intervention, the implementation team and partners were able to discuss the intervention’s design and the adaptations required (**design quality and packaging**). Still, it was difficult to foresee how the intervention would actually unfold, as it depended on the context and on the reactions and involvement of partners in the field. In this respect, one of the designers reflected that the intervention probably lacked clarity overall:

*“We had a fine theoretical idea*, *but how to materialize it concretely was less clear*. *If the idea*, *roles*, *and responsibilities had been clearer*, *and if the plan for activities and support had also been clearer*, *I think we would have had a better chance*.*”* (Design team member)

The ambiguity around what activities would be required to adapt to the needs of the actors is an indication of the intervention’s complexity (**complexity**), and this influenced both the process and the outcomes. Stakeholders’ needs for knowledge and RBE varied greatly (e.g. community health insurance, infectious diseases, health system financing, infant and child mortality, maternal health). In each case, the broker had to gather the theoretical content, develop user-friendly KT tools, and organize workshops to present and encourage the use of this knowledge. Furthermore, the fact that the intervention targeted a variety of health system actors, ranging from MHOs to health professionals and policymakers, added significantly to the complexity.

The financial resources allocated to the KB activities were limited, and the project was intended to run for a set period of four years (**cost**). Having only one full-time knowledge broker available, supervised by a senior broker, limited the strategy’s deployment and visibility. After the first year, the partners would have wanted more frequent activities to be conducted in the field. Members of the design and support teams pointed out that investing more resources might have motivated the KB team and resulted in more activities.

### Individuals

The researchers, design team, support team, and knowledge brokers had differing knowledge and beliefs about the importance of the intervention (**knowledge and beliefs about the intervention**). The implementation of such a strategy and its success in a new context appeared to have greater significance for the Canadian researchers. Some members of the implementation team did not participate in the activities as planned, and these deviations from the plan then created certain dissatisfactions in the team.

As the central actor in the innovation, expectations for the broker were high. He had to assume a role that was not yet very clear in the literature, and this, in a very short time frame. Thus, given his uncertainty about the tasks to be done, the development of his sense of competence was not optimal (**self-efficacy**). In a context that values social hierarchy, it can be difficult to simultaneously take on new responsibilities, develop new professional competencies, engage in networking, and inform key health system actors. The broker, whose training was almost exclusively in research, thus experienced a number of issues in adjusting to his role. For example, at the start of the intervention, the broker had no protocol to follow because everything needed to be constructed from scratch, in stark contrast to how research is conducted. This experience reaffirms that uncertainty associated with innovation implementation and the inherent difficulty to develop a work plan with precise steps can increase the feeling of discouragement and loneliness already associated with this intermediary role.

After two years of implementation, the broker believed in the need for his role in the Burkinabè context and was motivated to make the intervention a success. By then, he had made significant progress in terms of both interpersonal and technical skills (e.g. surveying the literature, drafting policy briefs, organizing deliberative workshops) (**skill proficiency**). However, certain shortcomings became apparent after the first years of implementation. For example, he had been expected to become more proactive in meeting regularly with partners. This lack of presence in the field weakened the links that had been established with partners at the start of the project. On that point, the broker points out that a greater presence could also have been perceived as intrusive for the RBE users, so that the optimal balance can be hard to reach. Other factors over which the broker had no control might also explain this difficulty, such as a lack of active support, absence of a leader or champion during implementation and difficulties related to receiving ongoing training at a distance. The broker's experience confirms the importance of providing a comfortable and functional physical work environment, but also a supportive psychological climate based on trust by the support team, to prevent him from feeling overly monitored and under pressure.

### Inner setting

Given the novelty of the brokering role and the fact that activities were to be developed based on stakeholders’ needs, an overall logic model for the intervention was developed beforehand. As such, brokering activities were defined generally but not operationalized concretely. Some reference guides were provided to the broker (e.g. literature search strategy), but for the relational component of his role, he was given no guidelines to follow (**access to knowledge and information**). For example, initiative and proactivity are important qualities in a broker. In this respect, the lack of close support from an onsite mentor who could provide real-time constructive feedback was a shortfall in the intervention (**goals and feedback**). As well, the respective roles and tasks of each team member, and what was expected of them, were not clearly defined at the outset (**formulation of tasks**). These points suggest the context was not entirely conducive to implementation of the intervention (**learning climate**). After the first two years of operation, certain deficiencies were noted in the intervention’s leadership and management (**leadership engagement**):

*“When you’re working as a broker*, *you need coaching and technical mentoring*, *but you also need someone to handle the political-strategic issues*, *a manager*, *and that wasn’t there…*.*”* (Support team member)

A common factor in all the above-mentioned difficulties was the poor quality of communications between the members of the implementation team (**networks and communications**). The lack of informal and formal exchanges about each person’s expectations and dissatisfactions with how the intervention was conducted reduced the chances of success. The notion of social hierarchy and fear of damaging professional collaborations might explain the fact that many things were left unsaid in the team.

Certain explanatory factors were also identified in relation to the contexts of the RBE users targeted by the KB activities. Before the intervention was launched, partnerships were established with district health system actors, and the strategy was tailored to the needs they expressed. Locally, KB activities were successful among MHOs, which recognized the value of the broker’s support in improving their practices. The organizational structure of these RBE users seemed to facilitate opportunities for informal exchanges with the knowledge broker by less bureaucratic procedures and greater decision-making authority. As such, the activities offered by the intervention were compatible with their needs (**compatibility**):

*“[The project] brought all the health mutuals to the table*, *where they could discuss health problems*, *develop points of view*, *and change intervention strategies*.*”* (MHO representative)

However, several questions remained about why the partners’ involvement was so low over the years. Although local stakeholders considered the KB activities to be useful, the implementation team observed that certain RBE users’ organizational cultures did not demonstrate an openness to change (**organizational norms regarding change**) or a perceived need to change after dissemination activities (e.g. stakeholders would rather favour a strategic use of RBE instead of enlightenment).

The KB intervention facilitated the partners’ access to RBE, provided them with meeting spaces and support in applying this knowledge, and all this with no financial incentives. At the beginning, this did not inhibit the partners’ attendance at local knowledge sharing workshops. However, several local partners mentioned the difficulty of obtaining operating funds for their organization, which may have limited their ability to apply the broker’s recommendations or to start new projects. The RBE users’ low involvement in the intervention over the long term might also have been linked to a lack of incentives provided by their organization (recognition, time allocated in their tasks, etc.) (**organizational incentives and rewards**).

As stated earlier, a lack of strategy’s ownership by the RBE users approached when scaling the activities was observed (**implementation readiness**). In addition to aspects related to organizational culture, certain factors seem to explain this, such as a difficulty in understanding the potential benefits and impact of KB activities as well as the absence of terms of reference for the collaboration process with the broker. At the central level, the ownership would have been greater if the intervention leadership and management were handled internally from the beginning. Moreover, the way the intervention operates differed from common practices known by local actors involved in development projects such as the absence of per diems for participating in dissemination activities and the expectation that stakeholders would play an active role in the intervention.

### Support system

From the start of the intervention, several training days were provided to the broker: two five-day onsite training sessions in January and May 2012, plus a two-week internship in Canada in organizations specialized in knowledge brokering (**training**). These helped strengthen and develop the broker’s skills in several areas: knowing the different roles of a knowledge broker, learning the steps involved in documentary research, becoming familiar with the process of supporting change, etc. Some training days were also provided between 2012 and 2013 to several of the broker’s potential partners, to familiarize them with KT and explain the role of a knowledge broker. However, several limitations were mentioned with respect to training that may have affected the broker’s sense of self-efficacy and his activities. For instance, the training sessions were very intensive and condensed because the trainers could not travel regularly to the field. It was clear that close monitoring was needed to support the broker properly and that continuous training was essential. The broker would have liked to have had training at different points during the intervention to be able to make course corrections as the work was being carried out. It would, no doubt, have been more effective to have an iterative feedback loop between the training sessions and concrete experiences with the partners:

*“People learn through action*: *they encounter issues*, *they need to discuss them with their peers*, *with their manager*.*”* (Support team member)

The broker was also accompanied by a support team comprised of Canadian KT specialists and an information management specialist. The team supported the broker in organizing his agenda, managing bibliographic references for documentary research, developing KB activities, designing policy briefs, assessing partners’ needs, etc. (**technical assistance**). Another element that may have negatively influenced the implementation was the fact that, in the beginning, the strategy’s designers also had the role of mentoring the broker. This role confusion may have limited authentic exchanges between the broker and those providing technical support. To a certain extent, the latter were also in a position of authority: the broker was supposed to report to them on the progress of activities, since they were responsible for overseeing the proper functioning of the strategy. This situation may have inhibited the broker’s expression of needs and shortcomings, such that the technical assistance provided was not optimal.

### Outer setting

Some obstacles might be partially explained by the social, political, or economic contexts of Burkina Faso. The KB activities, which began at the local level in one district, had generally been well received. The fact that partnerships were created with stakeholders as the intervention was being developed meant that it was designed to respond to their needs (**needs and resources**). For example, one district-level NGO representative spoke about the difficulty of accessing knowledge:

*“[We] were looking for tools to manage malnutrition at the community level*. *We needed to find out whether any tools had already been tried*, *and what the outcomes were*. *We always knew that what we wanted to do had already been documented*, *but we didn’t know where to find the results of those studies*.*”* (NGO representative)

To broaden the scope and the potential impact of the intervention, efforts were made to relocate the broker to the Ministry of Health during the second year of implementation. However, the implementation team had difficulty creating other external partnerships (**cosmopolitanism**). They were unable to capitalize on the senior broker’s professional relationships to get the KB activities transferred to the central level. With this in mind, one of the intervention’s partners raised, in the first year of implementation, the importance of promoting activities among the various health system stakeholders to enhance the intervention’s visibility:

*“The promoters need to make it [the KB intervention] known*. *A communication plan would need to be developed to get the attention of associations*, *NGOs*, *the government*, *etc*. *Of course*, *this would require resources*. *They need to convince ‘those people’ [of the value] of what they’re doing*.*”* (NGO representative)

Difficulties in accessing the central level thus limited the potential for concretely assessing whether the proposed KB activities could have responded to decision-makers’ needs (**needs and resources**). It should be noted that several years earlier, in 2009, a similar initiative had been implemented within the government by WHO. This initiative, *EvipNet*, had been accommodated at the Ministry of Health of Burkina Faso. The origin of that initiative (WHO), the substantial financial resources allocated, and the structure being housed directly in the Ministry of Health are all significant elements.

Also, opportunities to collaborate with political actors are neither frequent nor easily created. The intervention clearly did not have sufficient access to the political sphere. Furthermore, the political context was quite unstable during the implementation, with the popular uprising that led to the president’s departure in October 2014 and the setting up of a provisional government until the presidential elections in November 2015. These external contextual factors should also be taken into account. This period of instability in the different ministerial structures did not appear to be conducive to moving the broker into the government, nor to making RBE a priority in decision-making.

### Implementation process

Much effort was invested in planning the intervention in collaboration with partners (**planning**). However, the intervention occasionally had to be reoriented for several reasons: disengagement of certain partners; disagreement among researchers on the research results to be mobilized, lack of solicitation to create new partnerships in the Ministry, etc. (**executing**). The analysis revealed that having a detailed work plan that is prepared when the strategy is developed and updated over the course of the intervention would be useful to anticipate problems and identify solutions more rapidly. For instance, after the second year of implementation, a work plan was created. As one of the knowledge brokers had suggested, the aim of this plan was to better prepare the partners for the arrival of KB activities:

*“During the second year of implementation*, *the focus will need to be on medium-term planning*, *because the partners will need to see [the KB activities] coming*, *they’ll need to feel that something is happening and that it will benefit them*.*”* (Knowledge broker)

As mentioned earlier, the intervention planning workshops were attended by many health system actors. However, despite the relevance of the broker’s work and the partners’ interest at the outset, the design and support teams observed that the tasks of creating partnerships and maintaining them over time had been somewhat neglected (**engaging**). For example, the fact that several support team members were at a distance, combined with the absence of leadership on the ground, clearly constrained the ability to organize activities intended to explain the intervention’s objectives and mechanisms; also, the potential benefits to partners were not sufficiently well communicated.

While an implementation evaluation identified the activities carried out and their potential impacts, and the training sessions held at the start of the intervention were evaluated by participants, the implementation team believed that the lack of any regular evaluation of the work climate in the field and among team members was a shortcoming (**reflecting and evaluating**). For example, most communications in the implementation team were one-on-one, which greatly limited the circulation of information. Team meetings would have been useful for members to inform each other on progress made, the achievement of their respective objectives, and any concerns or dissatisfactions. One team member suggested that these types of reflexive exchanges might have enabled the team to re-adjust the intervention more rapidly.

## Discussion

The aim of the intervention implemented in Burkina Faso was to introduce the knowledge broker function into a program of interventional research in health. In creating partnerships with health system stakeholders in the intervention region, the broker was expected to assess their knowledge needs, survey the literature to identify the most recent research evidence, produce various KT tools tailored to the different stakeholders, and support them in applying this knowledge to improve their actions. Despite getting off to a slow start, the broker successfully supported the MHOs in the region, prepared several policy briefs, and organized a few KT workshops with NGOs. However, the lack of decisional power among local stakeholders and the absence of any organizational resources or incentives for involvement in the intervention seem to have hampered the implementation. The implementation team then tried to relocate the broker to the national level. Efforts to establish new partnerships and thereby broaden the scope of the intervention were ineffective. Furthermore, within the team, the lack of leadership in the field, the absence of a shared vision for reorienting the intervention, and poor communication limited the implementation’s chances of success. The positive effects observed were mainly at the local level in the district, following KB activities with MHOs and NGOs during the first years of implementation. Although the intervention also encountered obstacles at the district level, the difficulties encountered mainly came when the team attempted to relocate the intervention at the central level in order to have more impact. In summary, the KB activities seemed to respond to health system actors’ needs (i.e. better access to research-based evidence) but overall, the limited success of the intervention could be explained both by a lack of cohesion within the implementation team, lack of ownership of the intervention by local actors combined with organizational barriers limiting their engagement as well as an unstable political context, resulting in no window of opportunities to scale up the intervention at this period.

### Knowledge broker’s role

Taking on the broker role is not easy. Acquiring and adapting RBE, using various research dissemination techniques, and developing networks and partnerships are tasks requiring very different skills. Coming from the research field, the broker did not have all the expertise and skills that would have enabled him to be equally at ease communicating with researchers, managers, and decision-makers [10,22]. A recent study on the key characteristics of public health knowledge brokers in Kenya [43] noted, in fact, the importance of having policy experience, good knowledge of stakeholder networks, and an entrepreneurial attitude. The intervention would have benefited from allocating more time to the broker’s training and development in these capacities [12,44]. For example, the implementation could have been facilitated if more resources had been invested in creating French-language support tools for the broker and adapted to the intervention context: procedures for analyzing stakeholder networks; reflexive evaluation matrix; guide for the development of different KT products; plain language guidelines, guide for facilitating deliberative workshops; communications plan, etc.

### Importance of networks and leaders

The analysis also showed that several factors that played a role in the strategy’s failure corresponded to factors identified in the KB literature: the importance of establishing a relationship of trust with partners, of clearly defining the broker’s role in the team, and of providing adequate support [12,17]. Creating links with the many health system actors, continuously assessing their needs, and maintaining collaborations are time- and resource-intensive tasks [9,11,15]. These relational requirements and communication skills may have been underestimated in Burkina Faso during efforts to relocate and expand the intervention at the central level. In this regard, the use of strategic communication techniques and the real involvement of formal and informal leaders in the intervention are key elements to be promoted in KT strategies [45]. Strategic communication stresses the importance of interacting with partners in ways that are meaningful for them. The ways of informing them about the intervention, the language used, and the information transmitted will be different for each potential RBE user. Furthermore, leaders can engage, motivate, and sustain individuals in the intervention and are in a position to create changes in organizational cultures [46].

### Strategic partnerships and contextual challenges

Our experience raises questions about the potential impact of KT initiatives conducted and funded by researchers and affiliated with research facilities. We saw that it was particularly complex to integrate an external intervention into the national level. In the case of the present intervention, the team had only received a letter of support from the General Secretary of the Ministry of Health of Burkina Faso, the second-in-command at the Ministry. At that time, the research team decided to implement the intervention within a private organization that was one of the team’s partners, believing they would eventually be able to mobilize its considerable network of contacts to deploy the project. It seemed expedient to count on this partner’s long experience with the Ministry of Health and national organizations. Deeper analysis of the networks of health system actors in Burkina Faso and of the overall functioning of the decision-making process would have helped the designers to identify the best point of entry for the strategy [47]. A map of health system stakeholders can be helpful to better understand the interests, priorities, and motivations of each one [45]. The KB intervention’s implementation in Burkina Faso was confronted with higher-level challenges that are important to point here such as staff turnover in governmental structures, evidence-informed policy culture little developed at that time as well as a period of political instability in Burkina Faso during efforts to scale up the intervention, which left limited window of opportunity at that moment [48].

In future initiatives aimed at developing a KB function within government, it could be beneficial to build on existing structures and to use the human resources in place to develop a KT platform. However, the successful institutionalization of a structure within a ministry depends largely on the government’s political will to allocate the resources required for its functioning, as well as on the priority of the matter within government and the presence of a manager who displays strong leadership [49]. As such, more efforts will be needed to raise awareness of the importance of using RBE to inform policies in Burkina Faso. Since then, progress has been made on this issue with the establishment of a knowledge transfer and management unit within the Ministry of Health to support evidence-informed decision-making [38].

### Complexity of the system

The difficulties encountered by KT initiatives are not due only to characteristics of the intervention itself or to the relational issues experienced. They are often also caused by the complexity of the socio-political system within which they are operating [50]. Many factors come into play, such as the diversity of professional, organizational, and sectoral cultures present, as well as issues of power and politics among the actors [45]. For example, the fact that the KB intervention in this case was based on a North–South partnership probably influenced the implementation process. Without listing all the ethical issues involved in North–South partnerships, other issues that can apply to the KB intervention in Burkina Faso include many unspoken aspects of the partnership agreements, asymmetries in the collaborators’ responsibilities, the lack of transparency, and project designs often copied from a Western model [51,52]. Even though it may seem like common sense, this experience provides evidence to reaffirm that partnerships should not be taken for granted and that needs' assessment, imperative to change, shared vision and trust relationships should constantly be revisited and renegotiated even if it takes time.

### Lessons learned and recommendations

Based on the lessons learned from this experience, we can propose certain actions to be considered when developing and implementing any future KB strategy in Burkina Faso. Table 2 presents recommendations related to the constructs coming out of the conceptual frameworks used. These recommendations largely reiterate factors known to be associated with successful implementation. It appears important to restate these factors, as it remains difficult to put them into action when interventions are implemented in a real-world context.

**Table 2:** Key recommendations for knowledge brokering interventions.

STAGES	CONSTRUCTS	PROPOSED ACTIONS
**Intervention development**	Planning	Establish a process for planning KB activities that is sufficiently detailed to serve as a reference framework, while being adaptable to partners’ needs.
	Cosmopolitanism	Build on relationships with other organizations to ensure the intervention is known and securely embedded among partners.
	Needs and resources	Assess the partners’ needs and see whether the resources available in their organizations and those of the intervention can respond to those needs.
	Tension for change	Consider the need and readiness for change both in the intervention’s implementation team and among the partners.
**Partners engagement**	Engaging	Continually nurture and assess the partners’ engagement over the course of the implementation.
	Evidence strength and quality	Help the partners to recognize the importance of participating in knowledge brokering activities to improve their actions.
	Relative advantage	Assess partner organizations’ interests in investing time and resources in the intervention.
	Compatibility	Ensure that organizations integrate knowledge brokering and the use of research-based evidence into their organizational mission.
	Available resources Organizational incentives	Encourage organizations to offer incentives for their members to invest in and contribute to knowledge brokering activities.
**Implementation planning**	Relative priority	Ensure that the team agrees on the objective of the intervention and on each person’s level of involvement.
	Formulation of tasks	Define clearly the roles and responsibilities of team members in relation to the tasks to be accomplished.
	Leadership engagement	Ensure that the intervention’s leaders are fully involved, so that the strategy is implemented as planned and the broker is closely supervised.
	Goals and feedback	Continuously clarify expectations with the broker and provide constructive feedback to adjust activities in real-time.
	Skill proficiency	Reflect with the broker on the skills that need to be developed over the course of the implementation.
	Training	Provide training opportunities to perfect the broker’s skills and improve his/her sense of self-efficacy.
**Implementation process**	Networks and communications	Set up an open system of communication among team members.
	Champions	Get leaders from partner organizations involved in promoting the intervention by including them in the development of activities.
	Access to knowledge and information Design quality and packaging	Mobilize theoretical knowledge (political science, communications, leadership, social marketing, etc.) to develop potential solutions to issues encountered.
	Reflecting and evaluating	Engage in a process of reflection and evaluation over the course of the strategy’s deployment, with reference to the factors identified as essential to successful implementation.

When developing the intervention, it is essential to set up a planning process that includes, among other things, periodic assessments of certain key factors, such as potential partners’ needs and interests, their readiness for change, and the availability of enough resources to adequately meet the needs. We also recommend that sufficient effort be made to secure partners’ engagement, so as to ensure they will invest time and resources in the intervention and will be involved in making the strategy relevant and useful for them. In terms of the implementation within the team (brokers, designers, and support team), it is important that they have a common vision of the objectives and the activities to be conducted. We recommend that they develop a work plan together that explicitly sets out everyone’s roles and tasks, what is expected of each person, the skills to be developed over the course of the intervention, the specific objectives to be met, and the training to be provided to the knowledge broker. Lastly, to ensure the intervention is implemented as planned, it is important to put in place a process of continuously reflecting on and evaluating levels of partner engagement, the quality of the brokering activities, potential obstacles to the strategy’s deployment, etc.

### Limitations of the study

One important limitation of the study relates to the data available for analysis. Accessing certain data was difficult, not only because the evaluation was retrospective, but probably also because of the implementation problems reported in this article. It may be, therefore, that certain factors influencing the intervention are not adequately nuanced here. The first author, who was not involved in the intervention’s design or implementation, collected the observation data and analyzed the data set; this may have added an objective dimension to the post-intervention evaluation. Data on the external (social-political-economic) context of the intervention are also difficult to obtain. We therefore based some of our reflections on our collective experience in the field over the past decade in Burkina Faso. The generalizability of this case study is limited and further comparative studies with other similar KT initiatives are needed. However, this study was not aimed at building a comprehensive theory but instead to produce context-dependant knowledge about a real-world intervention implemented in a complex system. This case study contributes to the cumulative development of knowledge in the KT field in Burkina Faso and possibly in West Africa [53]. For example, the use of well-known implementation science frameworks allowed the identification of factors which may help understand other similar interventions and serve as guidance for the implementation process. However, although conceptual frameworks have deductively guided this study, the analysis was also open to constructs that could have emerged from the data.

## Conclusions

The termination of this KB intervention does not mean knowledge brokering is not relevant in Burkina Faso. More attention needs to be paid to planning the deployment of the intervention, to continuous skills development and communications among the KB team, and to periodic assessment of potential obstacles related to the complexity of the system within which the intervention has been implemented. We also recommend using conceptual frameworks from the field of implementation science, not only to plan the implementation of interventions, but also for evaluation. More studies are needed to draw general lessons from specific knowledge brokering experiences. This will help understanding how best to implement this type of KB intervention on the African continent and what is their potential effect on population health in the longer term.

## Supporting information

S1 Table Detailed chronology of knowledge brokering activities carried out. (DOCX)

S2 Table Domains of analysis and constructs. (DOCX)

S1 File Interview guides. (DOCX)
