# Supplementary material for: A health knowledge brokering intervention in a district of Burkina Faso: A qualitative retrospective implementation analysis
Source: PLoS One. 2019 Jul 26;14(7):e0220105. doi: 10.1371/journal.pone.0220105 (PMC6660220; doi:10.1371/journal.pone.0220105)
Supplement: S1 Table — (DOCX) [file pone.0220105.s001.docx]

**SUPPORTING INFORMATION**

S1 Table. Detailed chronology of knowledge brokering activities carried out

|  | YEAR 1  2012 | YEAR 2  2013 | YEAR 3  2014 |
| --- | --- | --- | --- |
| January | - Workshop to launch the research program and identification of research questions with partners | - Results dissemination workshop – Changing health behaviours - Follow-up meeting with MHOs - Drafting of a policy brief – Belonging to an MHO | - Follow-up meeting with HMOs - Drafting of a policy brief – Policies regarding subsidies and fees exemptions for healthcare services - Broker’s online newsletter #1 |
| February | - Attendance at a workshop on program evaluation - 1^st^ training – knowledge brokering | - Updating action plans with MHOs | - Drafting of a policy brief on the theory of self-determination applied to the use of bednets - Broker’s online newsletter #2 |
| March |  |  | - Preparation of a best practice guide for encouraging membership in MHOs - Broker’s online newsletter #3 |
| April |  | - Follow-up meeting with MHOs | - Broker’s online newsletter #4 |
| May | - 2^nd^ training – support processes - Field mission – partners |  | - Workshop to develop an action plan with MHOs - Discussion workshop based on evidence summaries - Broker’s online newsletter #5 |
| June | - Attendance at a workshop on establishing contact with partners - Field mission – updating partners’ questions |  | - Broker’s attendance at a conference - Broker’s online newsletter #6 |
| July |  | - Follow-up meeting with MHOs - Results dissemination workshop on bednets use | - Broker’s online newsletter #7 |
| August |  |  | - Broker’s attendance at a workshop on making presentations and creating partnerships - Deliberative workshop with MHOs - Broker’s online newsletter #8 |
| September | - Broker’s development internship in Canada | - Attendance at a symposium on the promotion of research evidence and innovations | - Broker’s online newsletter #9 |
| October | - Literature survey – MHO membership - Field mission - partners - Dissemination workshop on survey results | - Follow-up meeting with MHOs |  |
| November | - Field mission – following the dissemination workshop - Workshop to develop an action plan with MHOs | - Results dissemination workshop – Malaria project - Workshop on knowledge brokering |  |
| December | - Literature survey on changing health behaviours | - Preparation of a results dissemination plan – Malaria project |  |
