# Supplementary material for: A health knowledge brokering intervention in a district of Burkina Faso: A qualitative retrospective implementation analysis
Source: PLoS One. 2019 Jul 26;14(7):e0220105. doi: 10.1371/journal.pone.0220105 (PMC6660220; doi:10.1371/journal.pone.0220105)
Supplement: S2 Table — (DOCX) [file pone.0220105.s002.docx]

S2 Table. Domains of analysis and constructs

| DOMAINS | **CONSTRUCTS’ DEFINITION** |
| --- | --- |
| Intervention | **Adaptability -** The degree to which an intervention can be adapted, tailored, refined, or reinvented to meet local needs  **Intervention source -** Perception of key stakeholders about whether the intervention is externally or internally developed.  **Relative advantage -** Stakeholders’ perception of the advantage of implementing the intervention versus an alternative solution.  **Evidence strength & quality -** Stakeholders’ perceptions of the quality and validity of evidence supporting the belief that the intervention will have desired outcomes.  **Design quality & packaging -** Perceived excellence in how the intervention is bundled, presented, and assembled  **Complexity -** Perceived difficulty of implementation, reflected by duration, scope, radicalness, disruptiveness, centrality, and intricacy and number of steps required to implement  **Cost -** Costs of the intervention and costs associated with implementing that intervention including investment, supply, and opportunity costs. |
| Outer Setting | **Needs & resources -** The extent to which [local] needs, as well as barriers and facilitators to meet those needs are accurately known and prioritized by the organization (e.g., project and implementation team)  **External policy & incentives -** A broad construct that includes external strategies to spread interventions including policy and regulations (governmental or other central entity), external mandates, recommendations and guidelines, etc.  **Cosmopolitanism -** The degree to which an organization is networked with other external organizations. |
| Inner Setting | **Structural characteristics -** The social architecture, age, maturity, and size of an organization.  **Network & communications -** The nature and quality of webs of social networks and the nature and quality of formal and informal communications within an organization.  **Organizational culture -** Norms, values, and basic assumptions of a given organization  **-Organizational norms regarding change -** This refers to the collective reputation and norms held by an organization in relation to its willingness to try new approaches as opposed to maintaining the status quo  **-Shared decision-making -** The extent to which relevant parties collaborate in determining what will be implemented and how  **-Formulation of tasks -** Procedures that enhance strategic planning and contain clear roles and responsibilities relative to task accomplishments  **Implementation climate -** The absorptive capacity for change, shared receptivity of involved individuals to an intervention and the extent to which use of that intervention will be rewarded, supported, and expected within their organization.  **-Tension for change -** The degree to which stakeholders perceive the current situation as intolerable or needing change.  **-Compatibility -** The degree of tangible fit between meaning and values attached to the intervention by involved individuals, how those align with individuals’ own norms, values, and perceived risks and needs, and how the intervention fits with existing workflows and systems.  **-Relative priority -** Individuals’ shared perception of the importance of the implementation within the organization.  **-Organization incentives -** Extrinsic incentives such as goal-sharing awards, performance reviews, promotions, and raises in salary and less tangible incentives such as increased stature or respect.  **-Goals & feedback -** The degree to which goals are clearly communicated, acted upon, and fed back to staff and alignment of that feedback with goals.  **-Learning climate -** A climate in which: a) leaders express their own fallibility and need for team members’ assistance and input; b) team members feel that they are essential, valued, and knowledgeable partners in the change process; c) individuals feel psychologically safe to try new methods; and d) there is sufficient time and space for reflective thinking and evaluation.  **Readiness for implementation –** Tangible and immediate indicators of organizational commitment to its decision to implement an intervention  **-Leadership engagement -** Commitment, involvement, and accountability of leaders and managers with the implementation.  **-Available resources -** The level of resources dedicated for implementation and on-going operations including money, training, education, physical space, and time.  **-Access to knowledge & information -** Ease of access to digestible information and knowledge about the intervention and how to incorporate it into work tasks. |
| Individuals | **Knowledge & beliefs about the intervention -** Individuals’ attitudes toward and value placed on the intervention as well as familiarity with facts, truths, and principles related to the intervention.  **Self-efficacy -** Individual belief in their own capabilities to execute courses of action to achieve implementation goals.  **Individual identification with organization -** A broad construct related to how individuals perceive the organization (or the KB project) and their relationship and degree of commitment with that organization.  **Skill proficiency -** Possession of the skills necessary for implementation (e.g., literature review, interpersonal communication, workshop facilitation, etc.) |
| Support System | **Training -** Approaches to insure individuals proficiencies in the skills necessary to conduct the intervention and to enhance individuals’ sense of self- efficacy (e.g., knowledge broker)  **Technical assistance -** This refers to the combination of resources offered to individuals once implementation begins, and may include retraining in certain skills, training of new staff, emotional support, and mechanisms to promote local problem solving efforts |
| Implementation Process | **Planning -** The degree to which a scheme or method of behavior and tasks for implementing an intervention are developed in advance and the quality of those schemes or methods.  **Engaging -** Attracting and involving appropriate individuals in the implementation and use of the intervention through a combined strategy of social marketing, education, role modeling, training, and other similar activities.  **Executing -** Carrying out or accomplishing the implementation according to plan.  **Reflecting & evaluating -** Quantitative and qualitative feedback about the progress and quality of implementation accompanied with regular personal and team debriefing about progress and experience. |

Adapted from Damschroder et al. (2009) and Durlak & DuPre (2008).
