# Supplementary material for: A health knowledge brokering intervention in a district of Burkina Faso: A qualitative retrospective implementation analysis
Source: PLoS One. 2019 Jul 26;14(7):e0220105. doi: 10.1371/journal.pone.0220105 (PMC6660220; doi:10.1371/journal.pone.0220105)
Supplement: S1 File — (DOCX) [file pone.0220105.s003.docx]

**S1 File. Interview guides**

**Original version**

**ÉVALUATION DE L’IMPLANTATION**

Au cours de cet entretien, je vais vous poser quelques questions au sujet de la mise en œuvre des activités de courtage de connaissances réalisées depuis un an

Le but de l’entretien est de faire le point sur le projet pour l’améliorer de façon à ce qu’il réponde le plus possible aux besoins des intervenants et décideurs.

Tout renseignement fourni sera conservé de manière **confidentielle** par les enquêteurs.

Tout renseignement fourni sera traité et présenté de manière **anonyme**.

En tout temps vous pouvez cesser votre participation sans aucune justification.

Acceptez-vous que l’entretien soit enregistré sur support numérique ?

Avant de débuter, auriez-vous des questions ?

1) Quelques informations à votre sujet ?

- - Quel poste occupez-vous actuellement ?
  - Depuis combien de temps ?
  - Quels autres postes avez-vous occupé auparavant ?
  - Combien de temps dans le domaine lié à la santé ?

2) Pourriez-vous me décrire brièvement le mandat de votre organisation ?

3) Quelles sont vos responsabilités ?

4) Selon vous, comment les connaissances produites par les chercheurs pourraient-elles vous être utiles ?

5) Que connaissez-vous du projet de courtage de connaissances qui a été mis sur pied au début de l’année dernière ? À quelles activités avez-vous participé ?

6) Quelle est votre appréciation de ce projet ?

- - Répond-il à vos besoins ; si oui, donnez un ou des exemples ?
  - Quelles en sont les forces ?
  - Quelles en sont les limites ?

7) Que pourrait-on faire pour améliorer le projet (quelles sont les activités ou produits qui pourraient être développés) ?

8) Avez-vous d’autres commentaires à faire concernant les sujets abordés aujourd’hui ?

Nous vous remercions pour votre participation à ce projet. Votre temps et le partage de vos perceptions sont grandement appréciés.

**ÉVALUATION POST-IMPLANTATION**

Votre participation à cet entretien est sollicitée afin de mieux comprendre les différents facteurs qui ont influencé le déroulement du projet de courtage de connaissances au Burkina Faso. Vos perceptions sont primordiales pour prendre en compte le vécu subjectif de chacun dans cette analyse de l’avortement de la stratégie.

L’objectif de cet entretien n’est pas d’attribuer la faute à qui que ce soit, mais plutôt de participer à une réflexion d’équipe afin d’apprendre de cette expérience et ainsi pouvoir améliorer nos futures initiatives de transfert de connaissances

1. Quel a été votre rôle dans le projet de courtage de connaissances ?

2. Quel était le principal objectif de ce projet ?

3. Avec le recul, quelle est votre appréciation globale du projet ?

4. Croyez-vous que les activités de courtage de connaissances proposées étaient adaptées pour répondre à un besoin des acteurs en santé ?

5. Selon vous, qu’est-ce qui a pu représenter un obstacle à la réussite du projet au niveau de la planification et des caractéristiques du programme)?

**Processus de mise en oeuvre et soutien**

6. Selon vous, qu’est-ce qui a pu représenter un obstacle à la réussite du projet au niveau des communications au sein de l’équipe

7. Comment avez-vous perçu les relations entre les membres de l’équipe ? (entre les courtiers et les concepteurs/chercheurs par exemple)

8. Ce projet de courtage misait sur un partenariat Nord-Sud. Comment avez-vous vécu cette collaboration ?

9. Que pensez-vous de l’accompagnement et de la formation qui ont été offert au courtier ?

10. Pouvez-vous me parler de l’appréciation que vous faites du travail accompli par le courtier et de ses compétences personnelles ? Pouvez-vous me parler de l’appréciation que vous faites de l’apport du courtier sénior au projet ? (pour les courtiers) Avec le recul, quelle évaluation pouvez-vous faire de votre implication dans le courtage ?

11. Selon vous, qu’est-ce qui a pu représenter un obstacle à la réussite du projet au niveau du lieu de travail du courtier ?

12. Comment avez-vous perçu le climat de travail ? Ex : confiance, soutien à la prise de risques, espace de réflexion, etc.)

**Contexte organisationnel et externe**

13. Comment expliquez-vous qu’il a été difficile de créer des collaborations/partenariats avec les utilisateurs potentiels de la recherche ?

14. Selon vous, qu’est-ce qui a pu représenter un obstacle à la réussite du projet au niveau des acteurs/organisations ciblés par le courtage

15. Quelles actions auraient pu être entreprises pour mieux rejoindre les acteurs afin qu’ils connaissent mieux les bénéfices des activités de courtage ?

16. Selon vous, comment le contexte socio-politique a pu influencer le déroulement du projet ?

**Leçons apprises**

17. Concernant votre rôle dans le projet, qu’est-ce que vous feriez différemment si c’était à refaire ?

18. Quels sont les apprentissages clés à faire de cette expérience ?

Avez-vous d’autres commentaires à faire concernant les sujets abordés aujourd’hui ?

Nous vous remercions pour votre participation à ce projet. Votre temps et le partage de vos perceptions sont grandement appréciés.

**English version**

**IMPLEMENTATION PART**

*During this interview, I'm going to ask you a few questions about the first years of implementation of the knowledge brokering activities.*

*The purpose of the interview is to evaluate this project in order to improve it and ensure it meets your needs as much as possible.*

Preamble to participants:

| Any information provided will be kept confidentially by the research team.  Any information provided will be treated and presented anonymously. Your answers will be presented in such a way that you can not be identified  At any time you can stop your participation without any justification.  Do you agree that the interview be digitally recorded? |
| --- |

Before starting the interview, would you have any questions?

1) Background information:

🡪What is your position or role within your organization?

🡪For how long?

🡪What other positions have you held before?

🡪How many years of experience do you have in the health field?

2) Could you briefly describe the mandate of your organization?

3)What are your responsibilities?

4) In your opinion, how can research based evidence be useful in your work ?

5) What do you know about the knowledge brokering project that was initiated early last year?

What activities did you participate in?

6) Could you share your thoughts on this project ?

🡪Does it meet your needs; if yes, give one or more examples?

🡪What are the project strengths?

🡪What are its limits ?

7) What could be done to improve the project (what activities or products could be developed)?

8) Do you have any other comments you’d like to add in light of what we’ve been talking about?

Thank you for your input and participation in this project. Your time and insights are greatly appreciated.

**POST-INTERVENTION PART**

*Your participation in this interview is solicited to better understand the various factors that have influenced the implementation of the knowledge brokering project. Your perceptions are important for this post-implementation analysis to be comprehensive.*

*The goal is not to blame anyone, but rather to participate in a team reflection on what could have been done differently in order to learn from this experience and thus be able to improve our future knowledge translation initiatives.*

1. What was your role in the knowledge brokering project?

2. What was the main objective of this project?

3. Looking back, what is your overall appreciation of the project?

4. Do you believe that the knowledge brokering activities proposed met a need of the health actors?

5. In your opinion, what could have been an obstacle to the project's success in terms of planning and program characteristics?

**Implementation process and support team**

6.What could have been an obstacle to the success of the project in terms of communications within the team?

7. How did you perceive the relationships between the team members?

8. This knowledge brokering project was based on a North-South partnership. How did you experience this collaboration?

9. What do you think of the training and mentoring that was offered to the broker?

10. Can you tell me about your appreciation of the broker's work? Can you tell me about your assessment of the senior broker's contribution to the project? (for brokers) In hindsight, what assessment can you make of your involvement in the project?

11. In your opinion, what could have been an obstacle to the success of the project regarding the broker's place of work?

12. How did you perceive the work climate? (e.g. trust, support for taking risks, space for reflection, etc.)

**Inner and outer settings**

13. How do you explain that the project has had difficulty creating collaborations/partnerships with potential research-based users?

14. In your opinion, what could have been an obstacle to the success of the project at the level of the actors / organizations targeted by the KB activities?

15. What actions could have been taken to better reach out to the actors so that they better know the benefits of KB activities?

16. In your opinion, how did the socio-political context influence the project implementation?

**Lessons learned**

17. Regarding your role in the project, which things would you do differently if you had to do it again?

18. What are the key learnings from this KB experience?

Do you have any other comments you’d like to add in light of what we’ve been talking about?

Thank you for your input and participation in this project. Your time and insights are greatly appreciated.
